# A Social Determinant of Health May Modify Genetic Associations for Blood Pressure: Evidence From a SNP by Education Interaction in an African American Population

**DOI:** 10.3389/fgene.2019.00428

**Published:** 2019-05-10

**Authors:** Brittany M. Hollister, Eric Farber-Eger, Melinda C. Aldrich, Dana C. Crawford

**Affiliations:** ^1^Social and Behavioral Research Branch, National Human Genome Research Institute, National Institutes of Health, Bethesda, MD, United States; ^2^Vanderbilt Institute for Clinical and Translational Research, Vanderbilt University Medical Center, Nashville, TN, United States; ^3^Department of Thoracic Surgery, Vanderbilt Genetics Institute, Vanderbilt University Medical Center, Nashville, TN, United States; ^4^Department of Population and Quantitative Health Sciences, Cleveland Institute for Computational Biology, Case Western Reserve University, Cleveland, OH, United States

**Keywords:** electronic health records, social determinants of health, African Americans, blood pressure, gene-environment, education

## Abstract

African Americans experience the highest burden of hypertension in the United States compared with other groups. Genetic contributions to this complex condition are now emerging in this as well as other populations through large-scale genome-wide association studies (GWAS) and meta-analyses. Despite these recent discovery efforts, relatively few large-scale studies of blood pressure have considered the joint influence of genetics and social determinants of health despite extensive evidence supporting their impact on hypertension. To identify these expected interactions, we accessed a subset of the Vanderbilt University Medical Center (VUMC) biorepository linked to de-identified electronic health records (EHRs) of adult African Americans genotyped using the Illumina Metabochip (*n* = 2,577). To examine potential interactions between education, a recognized social determinant of health, and genetic variants contributing to blood pressure, we used linear regression models to investigate two-way interactions for systolic and diastolic blood pressure (DBP). We identified a two-way interaction between rs6687976 and education affecting DBP (*p* = 0.052). Individuals homozygous for the minor allele and having less than a high school education had higher DBP compared with (1) individuals homozygous for the minor allele and high school education or greater and (2) individuals not homozygous for the minor allele and less than a high school education. To our knowledge, this is the first EHR -based study to suggest a gene-environment interaction for blood pressure in African Americans, supporting the hypothesis that genetic contributions to hypertension may be modulated by social factors.

## Introduction

African Americans have a higher prevalence of hypertension, or chronically high blood pressure, compared with other racial/ethnic groups (Yoon et al., 2015; Writing Group Members et al., 2016). Despite this higher burden of disease in African Americans, early genome-wide association studies (GWAS) for hypertension and systolic blood pressure (SBP) and diastolic blood pressure (DBP) were limited to populations of European-descent (Levy et al., 2009; Newton-Cheh et al., 2009; Wang et al., 2009; International Consortium for Blood Pressure Genome-Wide Association Studies et al., 2011) or east Asian-descent (Kato et al., 2011). More recent GWAS have been performed in ancestrally diverse populations, including African Americans or African-descent populations (Adeyemo et al., 2009; Zhu et al., 2011, 2015; Kidambi et al., 2012; Franceschini et al., 2013; Hoffmann et al., 2017; Liang et al., 2017). Collectively, these associated common variants explain 3–6% of the variance for SBP and DBP, and in the largest European-descent study to date account for up to 27% of the estimated single nucleotide polymorphism (SNP)-wide heritability for these traits (Evangelou et al., 2018).

Current GWAS findings explain only a proportion of the expected contribution from additive genetic effects. Previous twin and family studies estimate these traits have moderate to high heritability (30–70%) (Fagard et al., 1995; Rotimi et al., 1999; Levy et al., 2000; Hottenga et al., 2005; Kupper et al., 2005), suggesting that additional genetic associations have yet to be discovered. Given that GWAS identify common single nucleotide variants (SNVs) for association, additional genetic associations may be found among rare SNVs (Doris, 2011; Russo et al., 2018). Importantly, most GWAS consider only main effects and do not consider interactions with relevant environmental exposures. Two recent and large GWAS of blood pressure have considered alcohol consumption (Feitosa et al., 2018) and smoking (Sung et al., 2018), both of which identified novel putative associations for these traits.

Here, we examine the modifying effects of education, a measure of socioeconomic status (SES) and recognized social determinant of health, on SBP and DBP traits among African Americans drawn from a clinical setting. Previous epidemiologic studies suggest that in addition to alcohol consumption and smoking, social environment and specifically SES has a strong influence on blood pressure and hypertension (Seeman et al., 2008; Cha et al., 2012; Non et al., 2012). Further, a GWAS in the Framingham Heart Study accounting for educational attainment identified novel associations for blood pressure traits among European Americans (Basson et al., 2014). Based on these prior findings, we hypothesized that educational attainment modifies associations between genetic variants and blood pressure among African Americans. To test this hypothesis, we accessed a large biobank linked to electronic health records (EHRs) in a racially diverse clinical population. We identified two associated SNPs, *ARHGAP22* rs4593967 (SBP) and *IQCK* rs950928 (DBP), neither of which has been previously associated with blood pressure. We also identified a novel SNP-education interaction affecting DBP, suggesting social determinants of health may modify genetic effects contributing to complex human traits.

## Materials and Methods

### Study Population and Data Collection

The study population is derived from BioVU, a DNA biobank of the Vanderbilt University Medical Center (VUMC) linked to de-identified EHRs. DNA samples are extracted from discarded blood samples drawn for routine clinical care (Roden et al., 2008). These samples are linked to the Synthetic Derivative (SD), the de-identified version of the VUMC EHR. Medical records within the SD are scrubbed of all Health Insurance Portability and Accountability Act (HIPAA) identifiers. This study was approved by the Vanderbilt University Institutional Review Board.

The study population consists of African American adults >18 years old drawn from a larger study of minority patients with DNA samples in BioVU (*n* = 15,863) (Crawford et al., 2015). We extracted relevant demographic variables, including race/ethnicity, sex, and age at data extraction available in the SD. Smoking status was extracted using International Classification of Diseases, Ninth Revision, Clinical Modification (ICD-9-CM) tobacco use codes as previously described (Wiley et al., 2013). Education was extracted from the free text of EHRs using a recently validated text-mining algorithm (Hollister et al., 2016). Education was modeled as a categorical variable: less than high school, high school, and some college or above. All weight and height measures were extracted from the EHR, and after extensive quality control, as described in Goodloe et al. (2017), median values were used to represent individual-level body mass index (BMI).

The median value of all blood pressure measurements within an individual’s EHR prior to a recording of blood pressure-altering medications in the patient’s medication list were used in analyses. Medications included in the keyword list of anti-hypertensives were angiotensin converting enzyme inhibitors, angiotensin II receptor blocker, beta blockers, non-dihydropyridine calcium channel blockers, hydralazine, Minoxidil, central alpha antagonists, direct renin antagonists, aldosterone antagonists, alpha antagonists, and diuretics including thiazides, K-sparing, and loop diuretics. Any blood pressure measurement found after any mention of these types of medications were excluded from analyses.

### Genotyping and Quality Control

Genotyping of 15,863 DNA samples from non-European descent individuals was performed using the Metabochip, a custom Illumina genotyping array designed to target SNPs and surrounding genomic regions associated with metabolic traits and cardiovascular disease (Buyske et al., 2012; Voight et al., 2012). We restricted the following quality control and statistical analyses to DNA samples from African Americans in BioVU (*n* = 11,301). All genotyping quality control was performed using PLINK 1.9 (Chang et al., 2015). After the removal of SNPs with a minor allele frequency of less than 5%, SNPs with a Hardy-Weinberg Equilibrium exact test *p*-value of less than 1 × 10^-7^, and SNPs with a genotyping call rate of less than 95%, a total of 115,834 variants remained (Supplementary Figure S1). We further removed 967 samples for either ambiguous sex, missing genotypes (>5%), or relatedness (twins, full siblings, parent/offspring) (Supplementary Figure S1). A total of 10,334 DNA samples passed genotyping quality control. After quality control, global ancestry was estimated using unsupervised ADMIXTURE analysis, assuming *K* = 2 (Alexander et al., 2009). Linkage disequilibrium (*r*^2^) was calculated using 1000 Genomes Phase 3 data and an expectation-maximization algorithm adapted from Haploview (Barrett et al., 2005) available through rAggr (Edlund et al., 2017).

Local ancestry for rs6687976 was assigned as previously described (Fish et al., 2018). Briefly, SHAPEITv2 (Delaneau et al., 2013) and the 1000 Genomes Phase 3 reference panel^1^ were used to phase the genotype data. RFMix (Maples et al., 2013) was used to assign local ancestry. Phased chromosomal haplotypes were matched to Yoruba and CEPH/European ancestral population panels from 1000 Genomes.

### Statistical Analysis

Inclusion criteria included African American adults with available Metabochip genotyping data and complete information on age, sex, BMI, premedication SBP, premedication DBP, smoking status, and education level. A total of 2,577 African Americans met genotyping quality control and had relevant covariates (Supplementary Figure S2). All statistical analyses were performed using PLINK 1.9 (Chang et al., 2015) or R (R Core Team, 2008). Linear regression models were used to identify genetic variants associated with either premedication SBP or premedication DBP. A Bonferroni adjusted *p*-value of 4.32 × 10^-7^ was used to determine significance. A main effects model included covariates for age, age squared, sex, BMI, smoking status, and percent global African ancestry:

$$\begin{array}{c} Premedication\;SBP\;or\;DBP=\;\beta_{0}+{\beta_{cov}}^{*}X_{cov}+{\beta_{1}}^{*}SNP+e \end{array}$$

A second main effects model included the same covariates, but also included education. To examine the interaction between genetic variants and education and how it may affect blood pressure, we modeled two-way interactions using a linear regression model and the same covariates as in our main effects model:

$$\begin{array}{c} Premedication\;SBP\;or\;DBP=\;\beta_{0}+{\beta_{cov}}^{*}X_{cov}+{\beta_{1}}^{*}SNP+{\beta_{2}}^{*}Education+{\beta_{3}}^{*}SNP^{*}Education+e \end{array}$$

The decision was made to focus on a set of SNPs which had a *p*-value of less than 1.4 × 10^-5^ from the main effects model to reduce issues with multiple testing. This significance threshold was chosen based on a Bonferroni correction for the number of SNPs that would remain if SNPs with an *r*^2^-value of greater than 0.1 were removed from our dataset. For this set of significant SNPs, we used a model which included the main effects of education and the SNP, as well as the interaction term between education and the genetic variants. The significance threshold for the interaction models was based on the number of SNPs tested for association with premedication SBP and DBP (*p* < 0.01 and *p* < 0.003, respectively).

## Results

### Population Characteristics

The final study population for analysis included 2,577 African American adults with Metabochip genotyping data and complete phenotype data (Supplementary Figures S1, S2). Among this study population, the majority were female (71%) with a median age of 38 years and median BMI of 26.8 kg/m^2^ (Table 1). Compared with a larger African American BioVU population genotyped on the Metabochip (Crawford et al., 2015), this subset had proportionally more females, was younger, and had a lower median BMI. The median premedication SBP and DBP were within the normal clinical range (122 and 74 mmHg, respectively) and most of the population was never smokers (87%; Table 1). The median percent global African ancestry was 81.7%. The majority of participants had at least a high school degree (Table 1). The median premedication SBP and DBP in this final study sample were statistically different (*p* < 0.05) from the larger study sample missing education data in the EHR but varied by only 3 mmHg (Supplementary Table S1).

**Table 1:** Study population characteristics representing African American adults from a biobank with electronic health record (EHR)-extracted blood pressure.

Characteristic	Number of individuals *n* = 2,577
Sex	
Male	753 (29%)
Female	1,824 (71%)
Median age in years (±SD)	38.2 (±15.4)
Median percent global African ancestry (range)	81.7% (1.0–99.9)
Education level	
Less than high school	328 (12.7%)
High school degree or equivalent	1518 (58.9%)
At least some college	731 (28.4%)
Smoking Status	
Never smokers	2,242 (87%)
Ever smokers	335 (13%)
Median body mass index (kg/m^2^; ±SD)	26.8 (±6.8)
Median premedication systolic blood pressure (mmHg; ±SD)	122.0 (±13.6)
Median Premedication diastolic blood pressure (mmHg; ±SD)	74.4 (±9.0)

*The population in this study was a subset of African Americans from the Vanderbilt University Medical Center (VUMC) biobank, BioVU. Samples were drawn from BioVU in 2011. All individuals had Metabochip genotype data which passed quality control measures. Individuals also had complete phenotype data which included age, sex, education level, smoking status, median body mass index (BMI), median premedication systolic blood pressure (SBP), and median diastolic blood pressure (DBP). These phenotypes were derived from the electronic health record. African ancestry was determined using ADMIXTURE. SD, standard deviation.*

### Predictors of Systolic and Diastolic Blood Pressure

In univariate analyses (Table 2), both premedication SBP and DBP were significantly associated with increasing age, male sex, and increasing BMI. SBP increased with age and DBP increased with age until around the age of 60, then began decreasing (Supplementary Figures S3, S4).

**Table 2:** Univariate analyses between relevant covariates and median premedication blood pressure values among African American adults.

Variable	Effect estimate, β (standard error)	*p*-value	Effect estimate, β (standard error)	*p*-value
	Premedication SBP		Premedication DBP	
Education level				
Less than high school	REF		REF	
High school degree and equivalent	0.16 (0.83)	0.84	1.95 (0.54)	0.0003^∗^
At least some college	1.26 (0.91)	0.17	2.54 (0.59)	<0.0001^∗^
Median age, years	0.31 (0.01)	<0.0001^∗^	0.19 (0.01)	<0.0001^∗^
Sex				
Male	REF		REF	
Female	–4.52 (0.52)	<0.0001^∗^	–1.48 (0.35)	0.0001^∗^
Median BMI (kg/m^2^)	0.38 (0.04)	<0.0001^∗^	0.25 (0.02)	<0.0001^∗^
Smoking status				
Never smoker	REF		REF	
Ever smoker	0.56 (0.72)	0.43	0.45 (0.48)	0.35
Median global African ancestry	–0.46 (1.89)	0.809	–1.24 (1.24)	0.32

*Prior to genetic analyses, covariates were examined to determine their association with the outcomes, premedication systolic (SBP) and diastolic blood pressure (DBP) in the study population, a subset of African Americans drawn from the Vanderbilt University Medical Center biobank BioVU (*n* = 2,577). Each linear regression model had either median premedication SBP or median premedication DBP as the outcome. The covariates included in each model were education level, median age, sex, body mass index (BMI), smoking status, and global African ancestry. Both premedication SBP and DBP were significantly associated with age, sex, and BMI. Premedication DBP is also significantly associated with education level. The symbol “^∗^” indicates statistical significance.*

Neither premedication SBP nor premedication DBP was associated with smoking status or global African ancestry. Also, education was not significantly associated with either premedication SBP or premedication DBP (Table 2 and Supplementary Figures S5, S6). Of all the variables tested, age and premedication DBP significantly co-varied with education (Supplementary Table S2).

### Education as a Modifier of Genetic Associations With Systolic and Diastolic Blood Pressure

To test for possible interactive effects between education and genetic variants associated with SBP and DBP, we examined three models: (1) initial single SNP tests of association without education as a covariate, (2) single SNP tests of association with education as a covariate, and (3) single SNP tests of association with SNP × education interaction terms. In the first model, single SNP tests of association were performed for SBP and DBP using linear regression adjusting for age, age squared, sex, BMI, smoking status, and percent global African ancestry. For both SBP (Supplementary Figure S7) and DBP (Supplementary Figure S8), only a single SNP was statistically significant using a Bonferroni correction (*p* < 4.32 × 10^-7^): *ARHGAP22* rs4593967 and *IQCK* rs950928, respectively.

The second set of models included education in addition to other relevant covariates (Supplementary Figures S9, S10). The addition of education to the model did not change the most significantly associated SNPs for either SBP or DBP (Table 3). In the regression model for SBP that included education, rs4593967 again passed Bonferroni correction (*p* < 4.32 × 10^-7^), and two other SNPs (rs10921895 and rs3804485) were associated at a suggestive significance threshold (*p* < 7.24 × 10^-6^). For DBP, rs950928 and rs8056711 passed Bonferroni correction. However, these SNPs have the same effect size and are in perfect linkage disequilibrium (*r*^2^ = 1.0), so they likely represent the same association.

**Table 3:** Characteristics of single nucleotide polymorphisms (SNPs) associated with premedication systolic and diastolic blood pressure with and without education in the model.

					Without education			With education		
SNP	Trait	Location	Gene	MAF	Effect	Standard	*p*-value	Effect	Standard	*p*-value
					estimate	error		estimate	error	
rs4593967	SBP	Intron	*ARHGAP22*	0.14	–2.51	0.46	1.45 × 10^-7^	–2.53	0.48	**1.16 × 10^-7^**
rs10921895	SBP	Intergenic		0.37	–1.52	0.37	5.31 × 10^-6^	–1.55	0.36	3.92 × 10^-6^
rs3804485	SBP	Intron	*LY86*	0.41	1.48	0.32	7.11 × 10^-6^	1.51	0.33	5.20 × 10^-6^
rs950928	DBP	Intron	*IQCK*	0.36	–1.04	0.24	3.13 × 10^-6^	–1.10	0.22	**4.53 × 10^-7^**
rs8056711	DBP	Intron	*IQCK*	0.36	–1.04	0.24	3.13 × 10^-6^	–1.10	0.22	**4.53 × 10^-7^**

*In the both sets of linear regression models, median premedication systolic blood pressure (SBP) and median premedication diastolic blood pressure (DBP) were the outcomes. Additionally, both sets of linear regression models included age, age squared, sex, median body mass index (BMI), smoking status, and median percent global African ancestry as covariates. The first set of models did not include education level. The second set of models included education. The addition of education to the model did not change which SNPs were most associated with SBP or DBP. Bolded *p*-values are considered statistically significant after Bonferroni correction.*

In the final set of models, education × SNP interaction terms were examined using SNPs associated with SBP or DBP at *p* < 1.4 × 10^-5^, as described above. No interaction terms met a strict Bonferroni correction (Supplementary Figures S11, S12). However, we identified a potential SNP-education interaction affecting DBP, rs6687976 (*p* = 0.052; Table 4). This potential interaction remained with the addition of local ancestry to the model. Individuals homozygous for the minor allele and having less than a high school education had higher DBP compared with (1) individuals homozygous for the minor allele and high school education or greater and (2) individuals not homozygous for the minor allele and less than a high school education (Supplementary Figure S13). No statistically significant interactions were identified for SBP (Table 4).

**Table 4:** Single nucleotide polymorphisms (SNPs) examined for interactions with education level impacting median premedication systolic and diastolic blood pressure.

SNP in education interaction term	*p*-value for the interaction between high school and SNP	*p*-value for interaction between college and SNP
**Systolic Blood Pressure**		
rs4593967_A	0.886	0.858
rs10921895_G	0.896	0.746
rs3804485_C	0.260	0.863
rs11066700_A	0.178	0.200
**Diastolic Blood Pressure**		
rs950928_G	0.175	0.298
rs8056711_C	0.175	0.297
rs9931167_A	0.927	0.503
rs11648873_T	0.940	0.478
rs28681321_A	0.941	0.461
rs6687976_A	**0.052**	0.996
chr16.19642355_G	0.094	0.110
rs3095994_A	0.738	0.726
rs1273518_G	0.218	0.648
rs1858975_A	0.076	0.091
rs4593967_A	0.512	0.768
rs8046628_C	0.181	0.316
rs6497402_A	0.062	0.085
chr16.19641087_A	0.064	0.076

*Median premedication systolic blood pressure (SBP) and diastolic blood pressure (DBP) were outcomes in the linear regression models. Covariates included in the models were age, age squared, sex, body mass index, smoking status, and African ancestry. The main effect of education and the SNP, as well as the SNP × education interaction term were also included in the model. Less than high school was the reference group within the regression models. The *p*-value for the potential SNP-education interaction is bolded.*

## Discussion

We sought to determine if education, a measure of SES and a recognized social determinant of health, modified genetic associations with SBP and DBP in African Americans. A previous study suggested gene × education interactions occur with blood pressure, but this study was conducted in a European-descent population (Basson et al., 2014). Associations between premedication SBP or premedication DBP and genetic variants from the Metabochip were examined, while including known predictors of blood pressure (age, BMI, sex, percent African ancestry, and smoking status) in the model. Results were compared with models which included a main effect for education, and a main effect for education plus a SNP-education interaction term. We observed a suggestive SNP by education interaction affecting DBP, a result not explained by local genetic ancestry. This potential interaction requires statistical replication and further investigation.

### Models Without Interaction

In univariate analyses the associations between premedication SBP and DBP and increasing age, male sex as well as increasing BMI were consistent with previous reports (August, 1999; Wright et al., 2011; Dua et al., 2014). The patterns of associations between SBP and DBP and age across the age continuum are also consistent with previous reports (Liang et al., 2017; Evangelou et al., 2018).

Intronic *ARHGAP22* rs4593967 was significantly associated with SBP and has not been previously reported as associated with blood pressure or hypertension. The minor allele frequency for *ARHGAP22* rs4593967 in this African American sample was 0.14, consistent with frequencies reported for African-descent populations included in The Genome Aggregation Database (0.148; Lek et al., 2016) and the 1000 Genomes Project (0.176; 1000 Genomes Project Consortium et al., 2015). Conversely, the minor allele is less frequently observed among populations of European (∼0.08) or East Asian-descent (<0.01). No other common (MAF > 1%) variants within 500 kb are in strong linkage disequilibrium (*r*^2^ ≥ 0.80) with rs4593967 in African-descent populations from the 1000 Genomes Project. *ARHGAP22* encodes the rho GTPase activating protein 22 and is widely expressed with highest expression levels in the brain. Variants within *ARHGAP22* have been associated with diabetic retinopathy, conduct disorder, daytime sleep, and self-employment (Dick et al., 2011; Huang et al., 2011; Van Der Loos et al., 2013; Spada et al., 2016), but these associations have not been replicated.

Intronic *IQCK* rs950928 was significantly associated with DBP after adjusting for multiple testing. Like *ARHGAP22* rs4593967, the minor allele frequency for *IQCK* rs950928 is higher among populations of African-descent (∼0.40) compared with European-descent populations (∼0.15). *IQCK* rs950928 is in perfect or strong linkage disequilibrium with rs8056711 and rs59009734 in African-descent populations, neither of which has been previously associated with human disease or traits. *IQCK*, which overlaps with several genes including *KNOP1*, encodes for IQ motif containing K and serves as an EF hand protein binding site. Like *ARHGAP22, IQCK* is highly expressed in the brain. A search within the Genotype-Tissue Expression (GTEx Consortium, 2013) database suggests that both rs8056711 and rs59009734 may be expression quantitative loci (eQTL), where each addition of the minor allele is associated with higher gene expression for several tissues including the right atrium auricular region of the heart and the aorta. While *IQCK* rs950928 and its associated SNPs rs8056711 and rs59009734 have not been previously associated with any phenotypes, common variants within *IQCK* have previously been associated with blood pressure, BMI, bone density, heart rate, chronic obstructive pulmonary disease, bipolar disorder, and a BMI-education interaction (Cho et al., 2009; Liu et al., 2010; Wan et al., 2011; Boardman et al., 2014; Winham et al., 2014).

Despite the present study’s small sample size (*n* = 2,577), there was sufficient power (80%) to detect significant associations with moderate effect size of 1.0 and a minor allele frequency of 0.20. For less common variants (MAF = 0.10), the study was powered to detect alleles with an effect size of 1.5 or greater. For variants with a MAF of 0.05, an effect size of 2.0 was needed in order to detect the variant’s effect. This study was not powered to detect any of the variants reported in the recent one million-person GWAS of blood pressure, as the variant with the largest effect size in that study was less than 1.0, with a median effect size of 0.219 mmHg (Evangelou et al., 2018). The limited power due to small sample size and limited directly genotyped variants likely contributed to the lack of replication of SNPs known to be associated with blood pressure in African Americans from previous GWAS.

### SNP × Education Interactions

We identified a possible SNP-education interaction affecting DBP for rs6687976 (*p* = 0.052). As the addition of local ancestry to the model did not alter the association, we expect that this observation is a result of true modifying effects of SES rather than ancestry. Individuals with two minor alleles and less than a high school education had higher blood pressure compared to those with two minor alleles and a high school education or those with less than a high school education and fewer minor alleles (Supplementary Figure S13). SNP rs6687976 is located within an intergenic region of chromosome 1 (Chr1:105674536 in GRCh37.p13) and has not been previously associated with any human traits within the literature. It is also not identified as an eQTL in GTEx (GTEx Consortium, 2013). Despite the limited information known about rs6687976, this result suggests that interactions between markers of social determinants of health and genetic variants affecting blood pressure likely exist, consistent with the findings of other studies that have observed interactions between genetic variants and social factors such as depression (Smith et al., 2017), perceived discrimination (Taylor et al., 2017), and cigarette smoking (Taylor et al., 2016).

### Limitations

The present study has several limitations. Primarily, the sample size is limited driven by the inclusion criteria of complete phenotype data for a specific racial/ethnic group within the larger clinical dataset. Therefore, we are unable to detect any variants of smaller effect sizes. The requirement for complete data may have also introduced biases that limit the interpretation and generalizability of these data.

In addition to the limited sample size, the study population was also different compared with previously published studies of blood pressure in African American populations. While the proportion of females, median BMI, percent African ancestry, median SBP, and median DBP were comparable with previous studies (Parra et al., 1998; Dumitrescu et al., 2015; Baharian et al., 2016; Franceschini et al., 2016; Jones et al., 2018; Restrepo et al., 2018), the population in this study did have a much lower median age, over 15 years younger. Given that blood pressure increases with age, this younger study population may have reduced variability in blood pressure measurements compared with the older published study populations with right-skewed distributions (Wright et al., 2011).

Another limitation was the lack of a replication dataset; therefore, all associations reported here are putative pending statistical replication or corroborative functional data. To date, other studies comparable or larger in sample size have not yet reported associations between these SNPs and blood pressure (Hoffmann et al., 2017). Furthermore, the genotyping array used here was also designed to include rare variation collected from the African ancestry samples as part of the 1000 Genomes Project. Therefore, many of the variants on the Metabochip were rare in African ancestry populations (Buyske et al., 2012) and filtered out during the quality control process as the present study was not powered to detect associations for rare variation.

There were also limitations regarding the phenotype data. All the variables were extracted from EHRs. While these records have extensive amounts of data, the data recorded by healthcare providers are not always accurate and the ability to extract the data can be limited. Furthermore although the positive predictive value of our algorithm was 80% (Hollister et al., 2016), there may have been inaccurate education information for the individuals within the dataset.

Determining which blood pressure measurements to use in the study is also a challenge, as measurements can vary widely across the EHR. The median blood pressure measurements were chosen for our study to reduce the influence of this variation. Beyond the inaccuracies and decisions to be made regarding the information within the EHR, blood pressure is difficult to measure within the clinic. Measurements of blood pressure can vary due to the calibration of instruments, the time of day it is measured, and due to illness (Jones et al., 2003). Patients also tend to have higher blood pressure within a clinical setting due to stress (Jones et al., 2003). To avoid these potential biases as much as possible, we chose median premedication blood pressure values for analysis, thereby avoiding outlier measurements and the changes introduced by blood pressure medications.

Finally, while education is a recognized social determinant of health, it is not a perfect proxy for social experiences. Still, evidence suggests that educational attainment can be a reflection of earning potential and social status (Shavers, 2007; Tamborini et al., 2015). Education has been shown to be associated with life expectancy, numerous biomarkers, and other health outcomes such as obesity and smoking (Seeman et al., 2008; National Center for Health Statistics, 2012). Low educational attainment itself is not the cause of poor health outcomes, but rather a variable often associated with individual-level behavioral determinants (e.g., smoking) or community-level determinants (e.g., racial segregation) that may influence blood pressure. Neither of these determinants is routinely recorded with the EHR; in contrast, educational attainment is often mentioned in the EHR. The availability of these data coupled with the observation that individual educational attainment is often stable over time make this variable a robust albeit imperfect proxy for social experiences.

### Strengths

Despite the limitations within the study, there were also several strengths. Primarily, this is the first study to incorporate EHR-derived education information into a large-scale genetic investigation. This study is a proof of principle that EHR-derived social determinant information can be investigated in a GWAS setting, thus breaking new ground to incorporate social factors in genetic studies among biobank populations. This is also the first analysis to observe an interaction between education and a common genetic variant with blood pressure in an African American population.

Despite the consistent association between social environment and health, social determinants of health are typically not included in genetic studies of health outcomes. For studies that access biobanks, the lack of social determinant data is likely related to the difficulty in accessing these data within the EHR, where they are not usually recorded in structured fields. The algorithms used in our study are the first to extract these important data from EHRs for research purposes (Hollister et al., 2016).

This study paves the road for the incorporation of education, as well as other social determinants of health, into genetic studies using biobank populations. The SNP-by-education interaction we observed affecting DBP (rs6687976) could suggest an example of a possible biological impact of the adversity experienced due to lower educational achievement. Only individuals homozygous for the minor allele who had less than a high school education experienced an increase in DBP. This association needs to be replicated; however, it suggests a potential pathway for the biological imbedding of stress experiences (represented by lower educational attainment) affecting blood pressure and risk for hypertension. Further studies are needed to support this hypothesis. We anticipate that this research will encourage other investigators to continue to study the genetics of health outcomes associated with racial health disparities and to incorporate social determinants of health within these studies.

## Author Contributions

BH conducted the analyses and wrote manuscript. EF-E helped to extract the phenotype data from the electronic health record. MA and DC contributed to guidance on project, and manuscript writing and editing.

## Conflict of Interest Statement

The authors declare that the research was conducted in the absence of any commercial or financial relationships that could be construed as a potential conflict of interest. The handling Editor declared a past co-authorship with one of the authors DC.

## Footnotes

1 https://mathgen.stats.ox.ac.uk/impute/impute_v2.html#reference
