# Supplementary material for: A Social Determinant of Health May Modify Genetic Associations for Blood Pressure: Evidence From a SNP by Education Interaction in an African American Population
Source: Front Genet. 2019 May 10;10:428. doi: 10.3389/fgene.2019.00428 (PMC6523518; doi:10.3389/fgene.2019.00428)
Supplement: Supplementary file 1 [file Data_Sheet_1.pdf]

Supplementary Material

1 Supplementary Figures and Tables

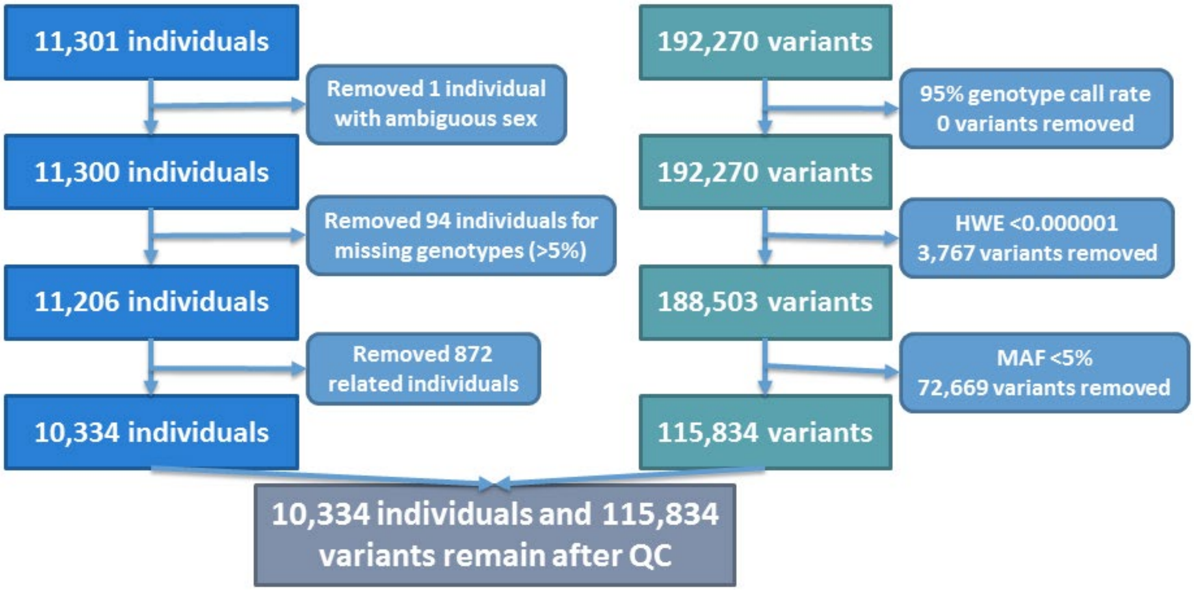

Figure S1. **Genotype quality control procedures for full dataset.**

Of the 15,863 DNA non-white BioVU samples genotyped using the Illumina Metabochip, 11,301 DNA samples were from African Americans, identified through administratively reported race in the electronic health record. We performed standard genotyping quality control for both the variants assays and the DNA samples genotyped. Shown here are the quality control thresholds or reasons for exclusion from further study and the associated counts.

Abbreviations: Hardy Weinberg Equilibrium (HWE), Minor Allele Frequency (MAF), Quality Control (QC).

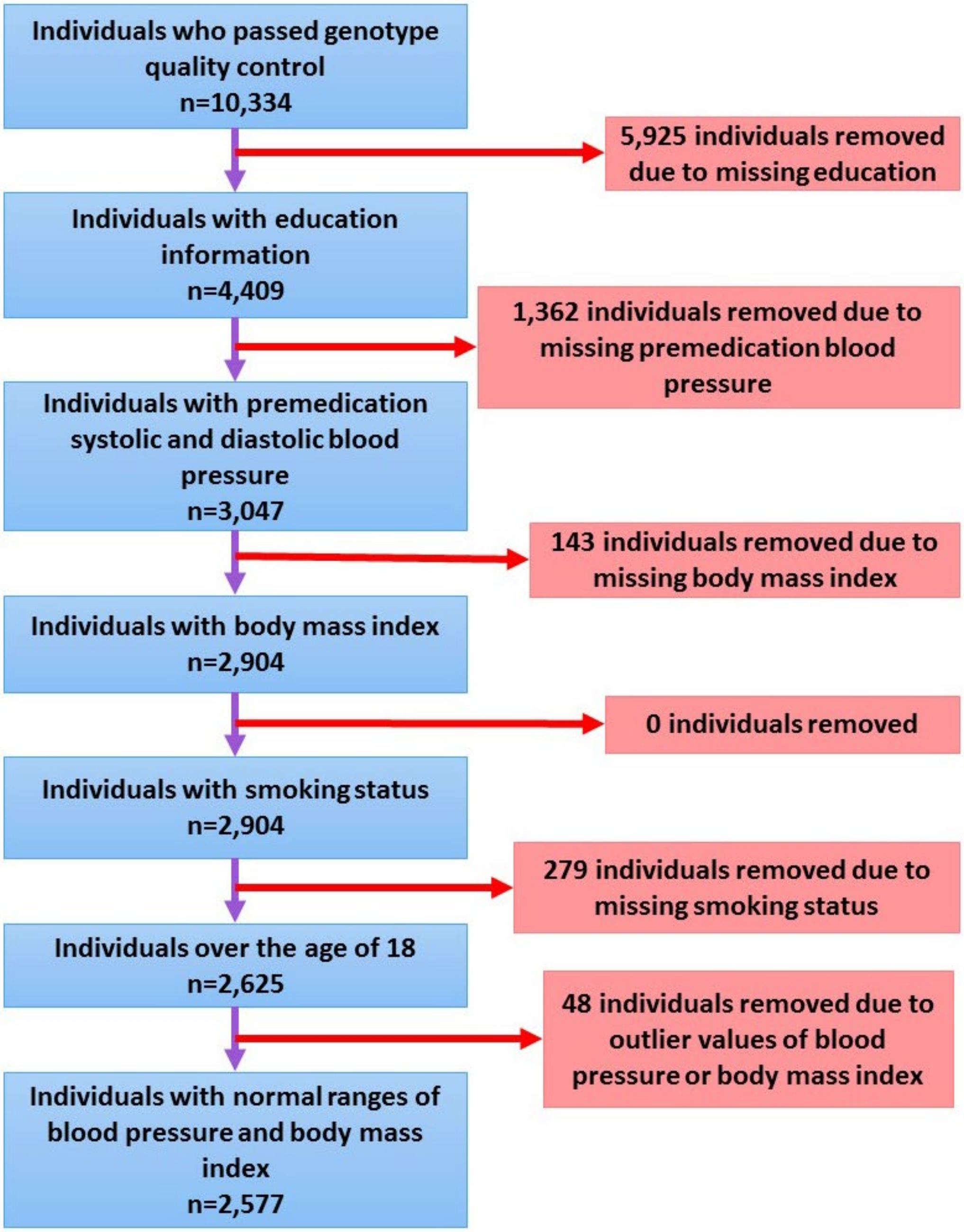

Figure S2. **Phenotype data quality control procedure.**

After conducting standard genotype quality control procedures, we had a dataset of 10,334 individuals. Individuals with missing phenotype data in the electronic health record were removed from the dataset. We also removed genotyped individuals <18 years of age. We defined normal range of blood pressure and body mass index as a value within three times the standard deviation. Individuals outside this range were removed from further analysis. From among 10,334 DNA samples from African Americans genotyped on the Metabochip, a total of 2,577 African American adults with Metabochip data remained after removal of samples based on absence or out-of-range phenotype and covariate data.

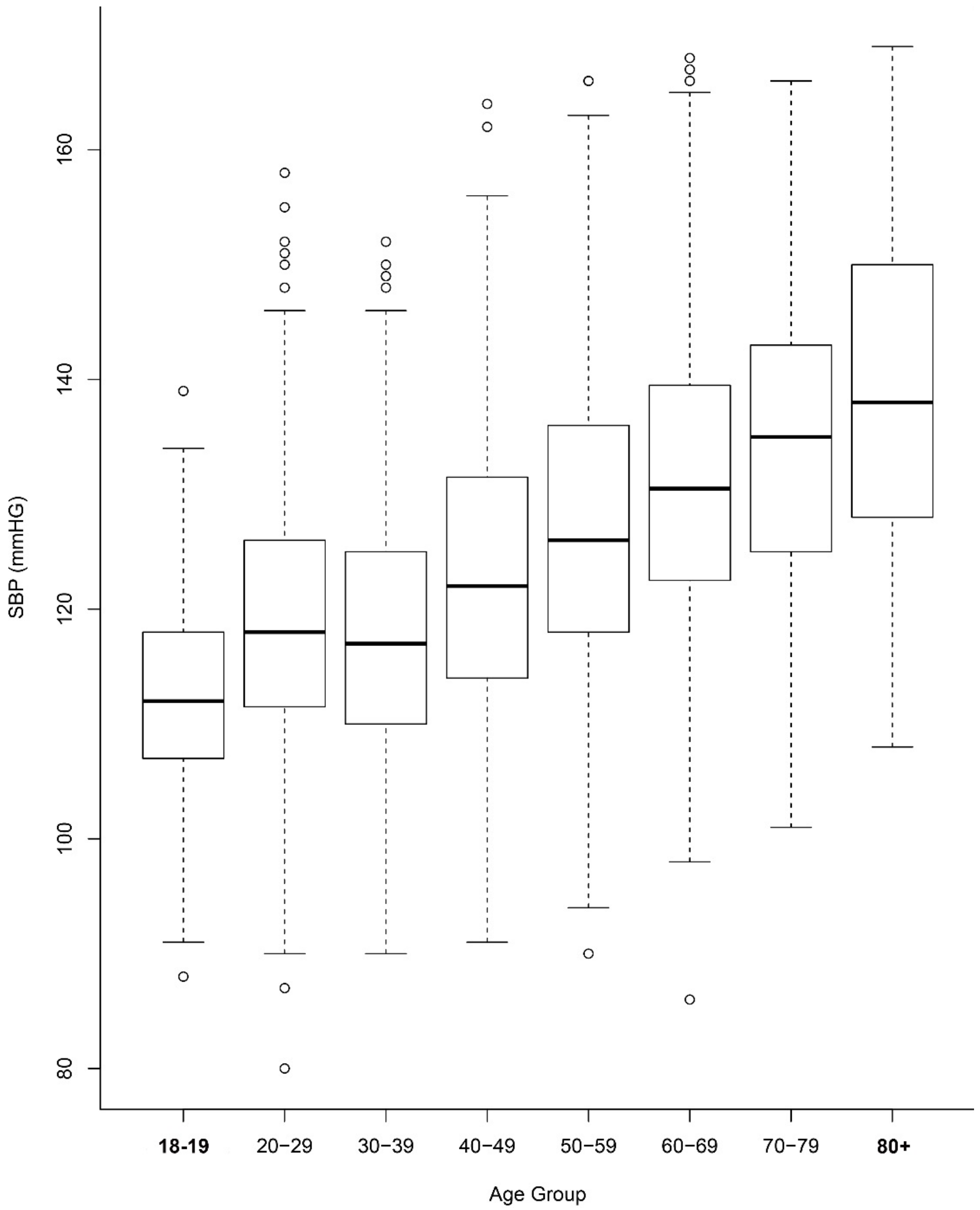

Figure S3. Premedication systolic blood pressure increases with age within the study population.

Premedication systolic blood pressure increases across age groups within our study population of African Americans from BioVU (n=2,577), as expected. The x-axis shows median participant age, grouped by decade. The y-axis shows the median premedication systolic blood pressure.

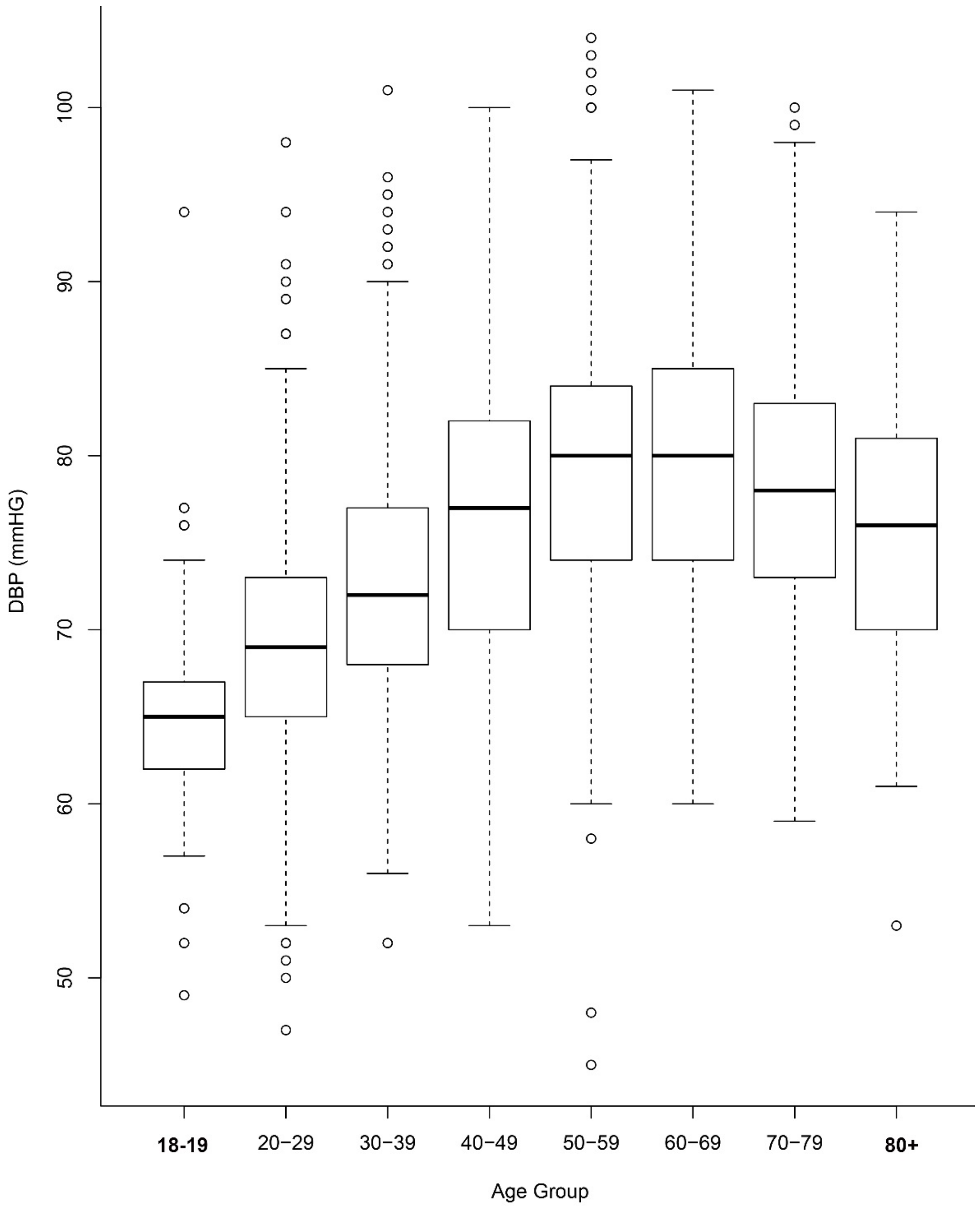

Figure S4. Premedication diastolic blood pressure increases with age until later in life within the study population.

Median premedication diastolic blood pressure increases until around age 60, then decreases in older individuals within our study population of African Americans from BioVU (n=2,577). The x-axis shows median participant age, grouped by decade. The y-axis shows the median premedication diastolic blood pressure.

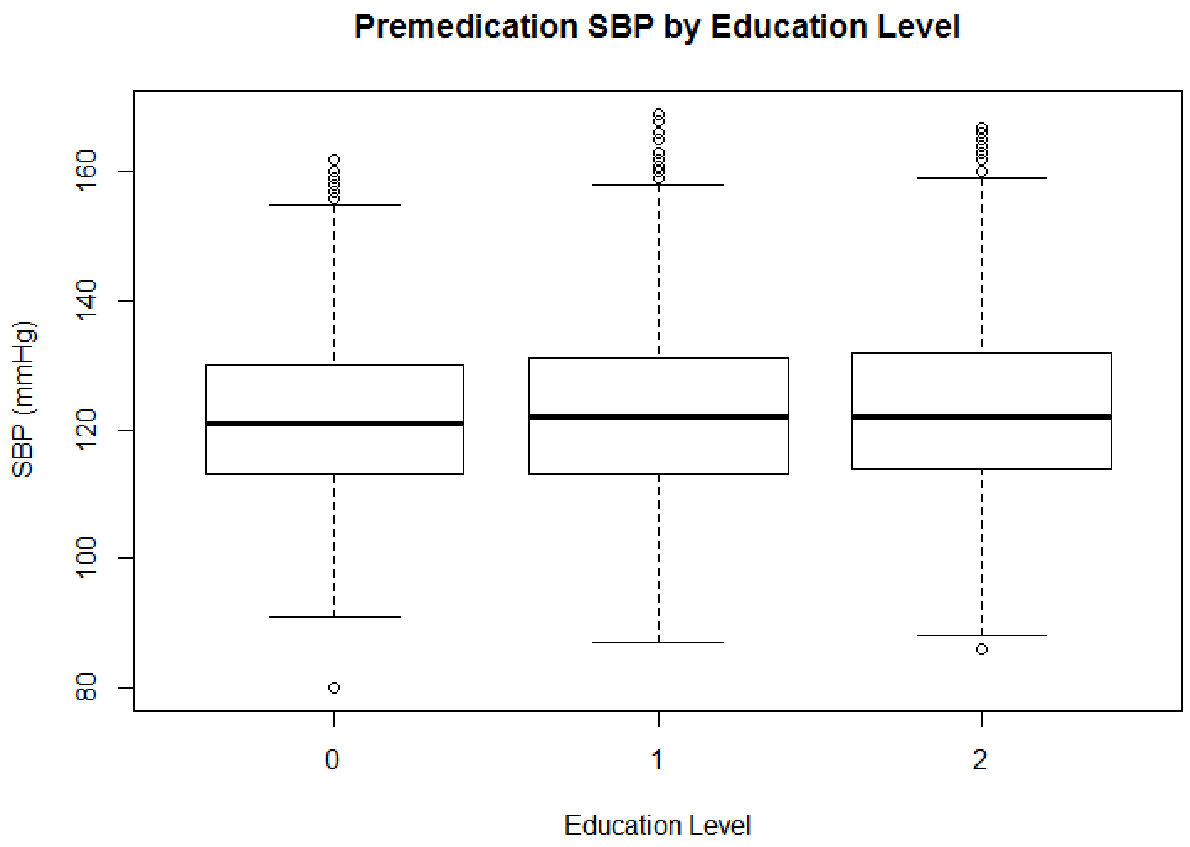

Figure S5. Premedication systolic blood pressure by education level within the study population.

Median premedication systolic blood pressure was not associated with education level within our study population of African Americans within BioVU (n=2,577). The x-axis shows education level: Level 0 indicates less than high school, level 1 indicates high school degree or equivalent, level 2 indicates some college and above. The y-axis shows the median premedication systolic blood pressure.

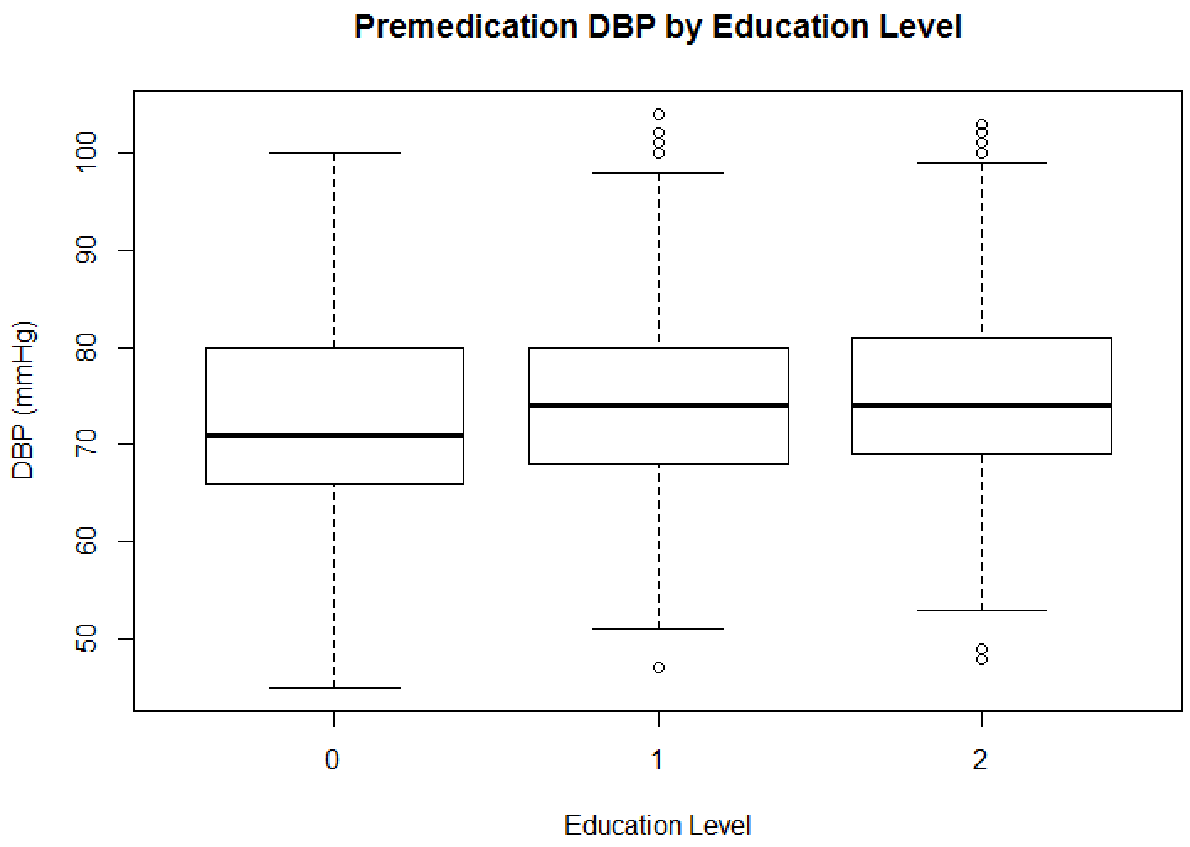

Figure S6. Premedication diastolic blood pressure by education level.

In a linear regression model predicting premedication diastolic blood pressure, education level was significantly associated with the outcome: high school or equivalent (β=1.95, SE=0.54, p=0.0003) and some college and above (β=2.54, SE=0.59, p<0.0001). Median age, sex, median body mass index, smoking status, and median African ancestry were included as covariates in the model. The x-axis shows education level: Level 0 indicates less than high school, level 1 indicates high school degree or equivalent, level 2 indicates some college and above. The y-axis shows median premedication diastolic blood pressure.

Variable Name	Included in analyses (n=2,577)	Individuals missing education (n=5,925)
Sex*		
Male	753 (29%)	2,173 (37%)
Female	1,824 (71%)	3,752 (63%)
Age* Median years	38	57
Median global African ancestry	81.7%	81.7%
Smoking status*		
Ever smokers	335 (13%)	795 (14%)
Never smokers	2,242 (87%)	4,295 (72%)
Missing	0 (0%)	835 (14%)
Body mass index* Median kg/m^2	26.8	27.8
Premedication SBP* Median mmHg	122	125
Premedication DBP* Median mmHg	74	77

* indicates $p < 0.05$

Table S1. Comparison of population characteristics between individuals includes in analyses and individuals excluded due to missing education information.

Differences where tested using t-test for age, global African ancestry, body mass index, premedication systolic blood pressure (SBP), and premedication diastolic blood pressure (DBP). Chi square tests were used for sex and smoking status. While there are statistically significant differences between sex, age, smoking status, body mass index, premedication SBP and premedication DBP, the most striking difference is the median age between groups.

**Variable**	**Degrees of freedom**	**Sum of squares**	**Mean squares**	**F value**	**p-value**
Age	1	8874	8874	28.45	**$1.05x10^{-7}$**
Sex	1	0.6	0.57	2.74	0.098
Smoking status	1	0.0	0.003	0.02	0.879
BMI	1	154	154	3.31	0.069
Premed SBP	1	545	545	2.94	0.087
Premed DBP	1	1221	1221.1	15.3	**$9.4x10^{-5}$**

Table S2. Analysis of covariance (ANCOVA) between three level education variable and blood pressure, age, sex, smoking status, and body mass index.

Education is the independent variable and the other variables are examined individually, to see if they covary with age, without the other variables in the model. Education significantly co-varies with age and premedication diastolic blood pressure.

Abbreviations: Systolic Blood Pressure (SBP), Diastolic Blood Pressure (DBP)

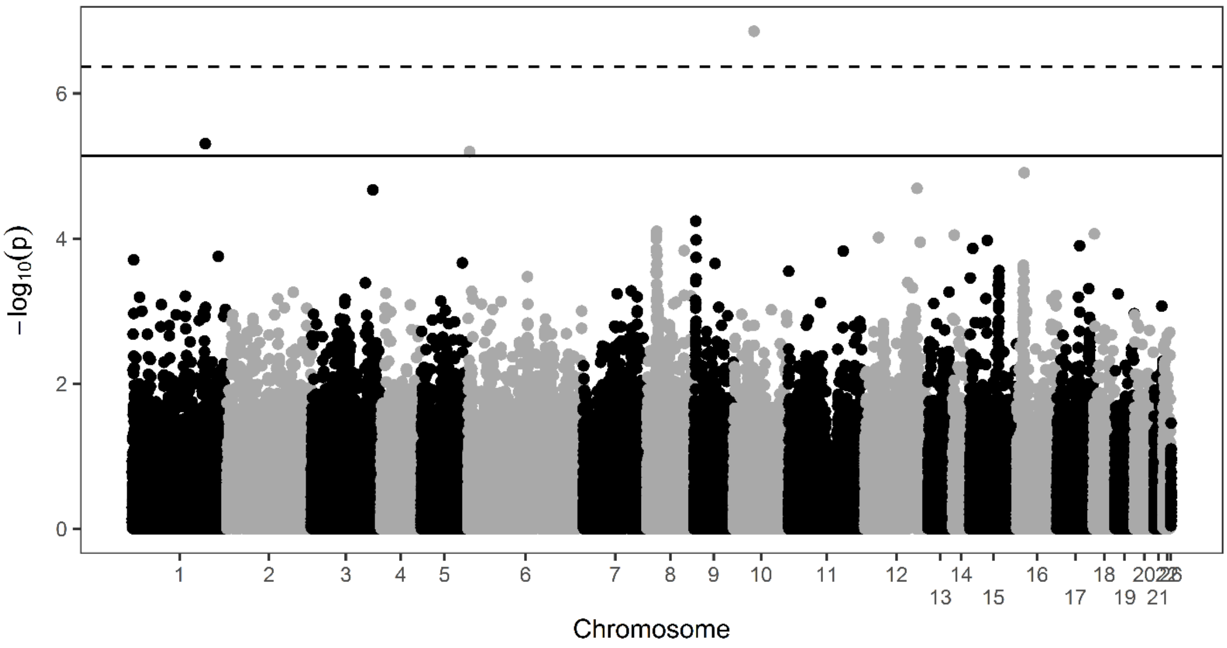

Figure S7. Manhattan plot of premedication systolic blood pressure without education included in the model.

Analysis of SNPs associated with median premedication systolic blood pressure was conducted in an African American population from Vanderbilt's biobank, BioVU (n=2,577). Covariates included in the linear regression model of median premedication systolic blood pressure included age, age squared, sex, body mass index, smoking status, and African ancestry. The x-axis shows SNP position grouped by chromosome number. The y-axis shows the -$\log_{10}$ of the p-value for the SNP, which indicates that smaller p-values are higher on the axis. The dashed line is a Bonferroni correction, a p-value of 4.32×10^{-7}. The solid line is a suggestive line, which was calculated by removing SNPs with an r^2 of higher than 0.6, was 7.24×10^{-6}. The SNP most significantly associated was *ARHGAP22* rs4593967, which has not been previously associated with systolic blood pressure.

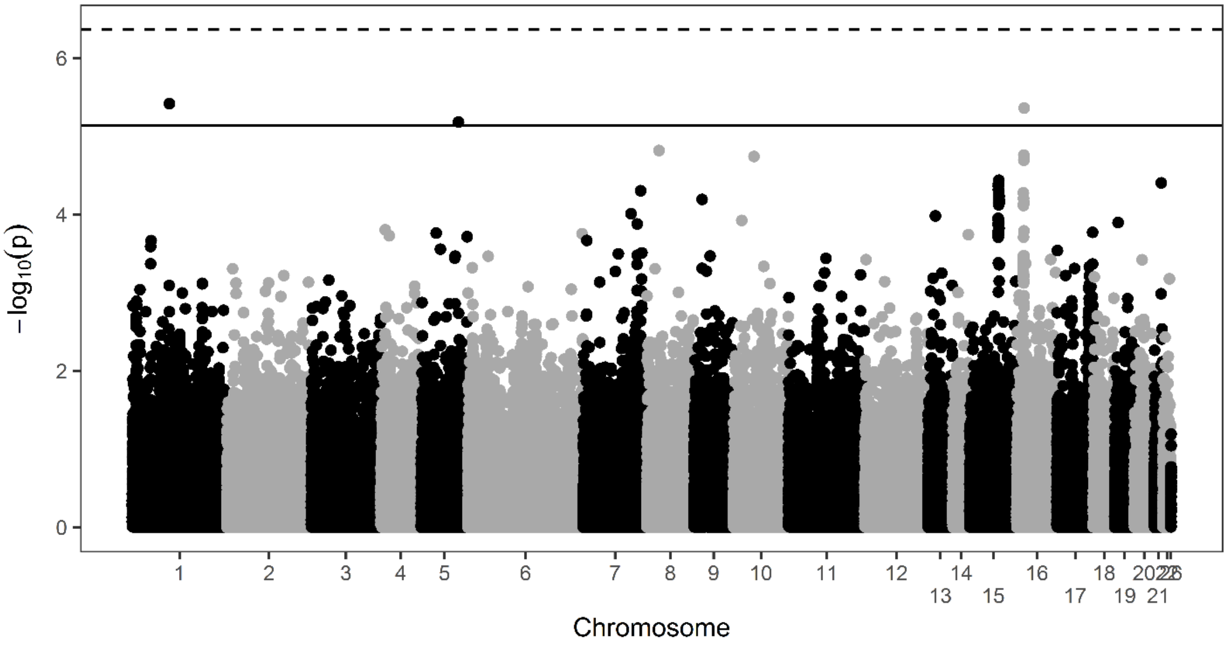

Figure S8. Manhattan plot of premedication diastolic blood pressure without education included in the model.

Analysis of SNPs associated with median premedication diastolic blood pressure was conducted in an African American population from Vanderbilt's biobank, BioVU (n=2,577). Covariates Covariates included in the linear regression model of median premedication diastolic blood pressure included age, age squared, sex, body mass index, smoking status, and African ancestry. The x-axis shows SNP position grouped by chromosome number. The y-axis shows the $-\log_{10}$ of the p-value for the SNP, which indicates that smaller p-values are higher on the axis. The dashed line is a Bonferroni correction, a p-value of 4.32×10^{-7}. The solid line is a suggestive line, which was calculated by removing SNPs with an r^2 of higher than 0.6, was 7.24×10^{-6}.

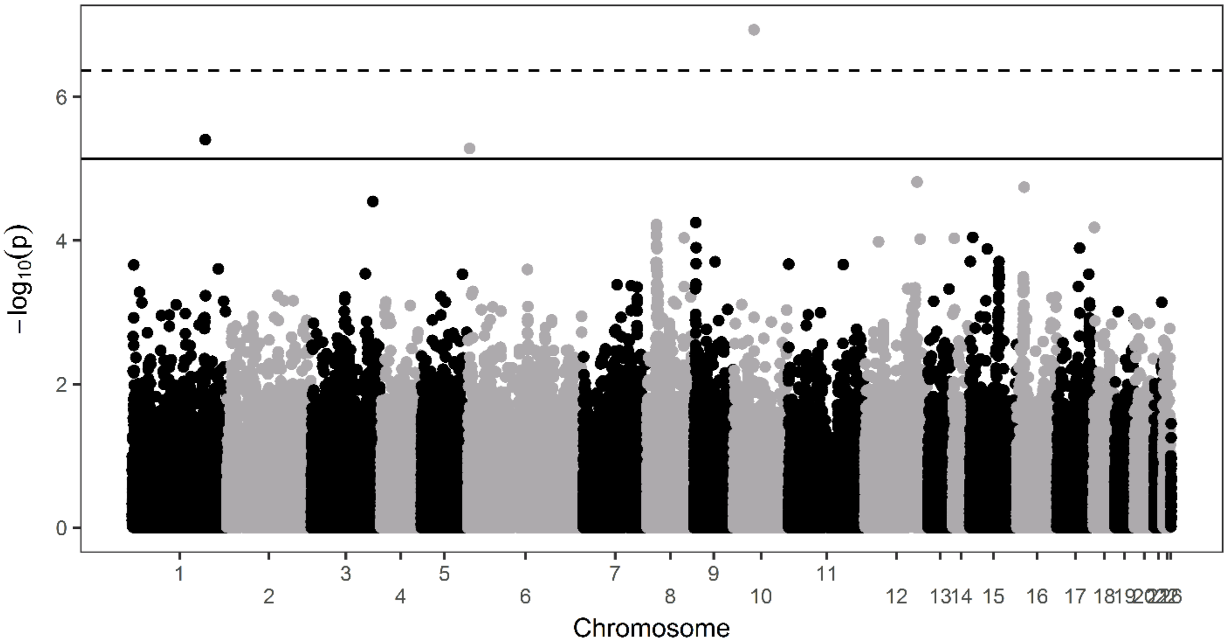

Figure S9. Manhattan plot of premedication systolic blood pressure with education included in the model.

Analysis of SNPs associated with median premedication systolic blood pressure was conducted in an African American population from Vanderbilt's biobank, BioVU (n=2,577). Covariates included in the linear regression model of median premedication systolic blood pressure included age, age squared, sex, body mass index, smoking status, and African ancestry. Education is included in this model, coded as less than high school, high school and equivalent, and some college and above. The x-axis shows SNP position grouped by chromosome number. The y-axis shows the $-\log_{10}$ of the p-value for the SNP, which indicates that smaller p-values are higher on the axis. The dashed line is a Bonferroni correction, a p-value of 4.32×10^{-7}. The solid line is a suggestive line, which was calculated by removing SNPs with an r^2 of higher than 0.6, was 7.24×10^{-6}. The addition of education did not change which SNP was most significantly associated, *ARHGAP22* rs4593967, which has not been previously associated with systolic blood pressure.

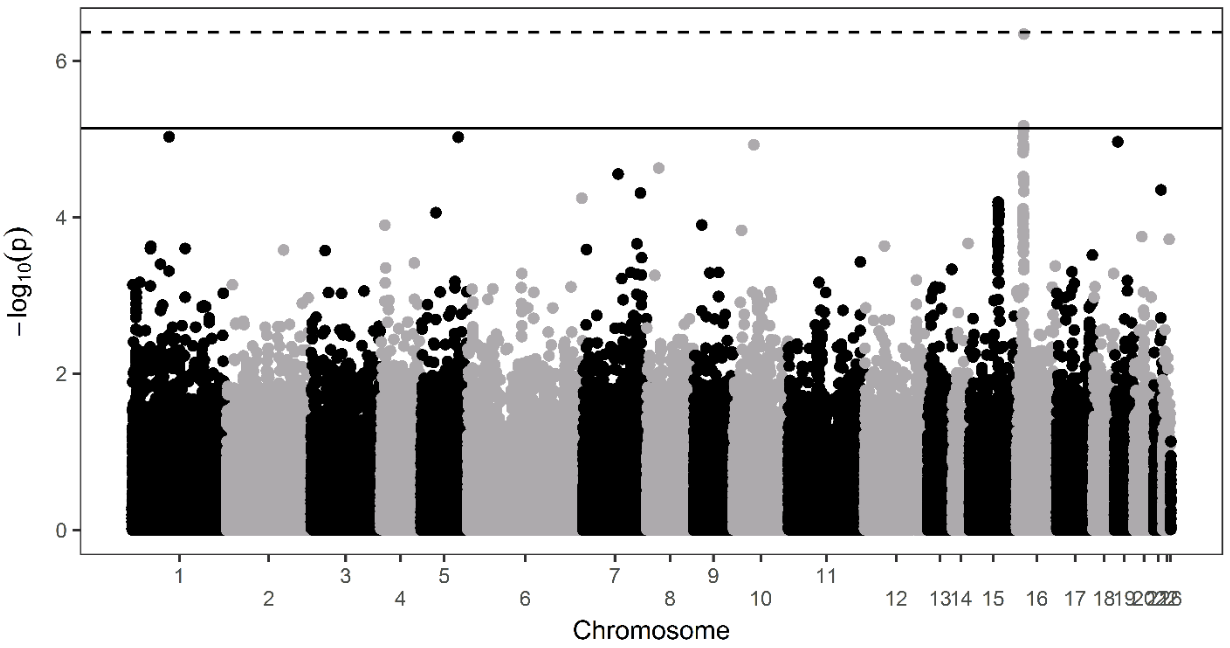

Figure S10. Manhattan plot of premedication diastolic blood pressure with education included in the model.

Analysis of SNPs associated with median premedication diastolic blood pressure was conducted in an African American population from Vanderbilt's biobank, BioVU (n=2,577). Covariates included in the linear regression model of median premedication diastolic blood pressure included age, age squared, sex, body mass index, smoking status, and African ancestry. Education is included in this model, coded as less than high school, high school and equivalent, and some college and above. The x-axis shows SNP position grouped by chromosome number. The y-axis shows the $-\log_{10}$ of the p-value for the SNP, which indicates that smaller p-values are higher on the axis. The dashed line is a Bonferroni correction, a p-value of 4.32×10^{-7}. The solid line is a suggestive line, which was calculated by removing SNPs with an r^2 of higher than 0.6, was 7.24×10^{-6}. With the addition of education, *IQCK* rs950928 is now statistically significant. It has not been previously associated with diastolic blood pressure.

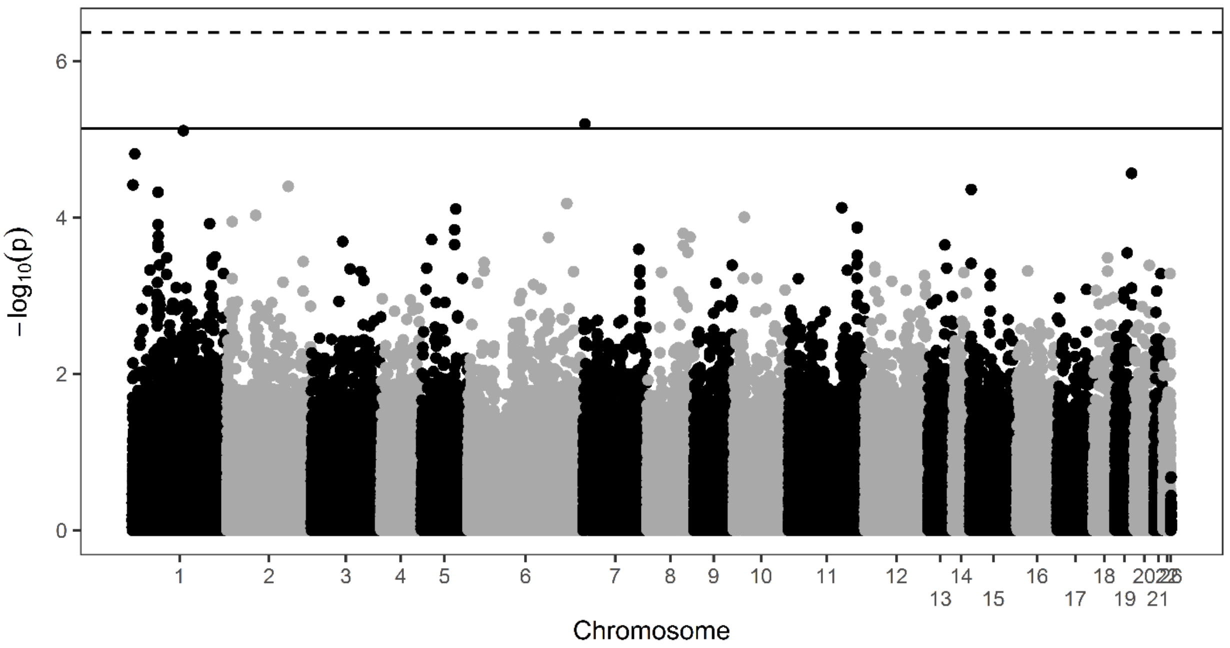

Figure S11. Manhattan plot of SNP x education interaction term p-values for premedication systolic blood pressure linear regression analysis.

Analysis of SNPs associated with median premedication systolic blood pressure was conducted in an African American population from Vanderbilt's biobank, BioVU (n=2,577). Education and SNP main effects, as well as SNP x education interactions were included in this model. Education is coded as less than high school, high school and equivalent, and some college and above. Covariates included in the linear regression model of median premedication systolic blood pressure included age, age squared, sex, body mass index, smoking status, and African ancestry. The x-axis shows SNP position grouped by chromosome number. The y-axis shows the $-\log_{10}$ of the p-value for the SNP, which indicates that smaller p-values are higher on the axis. The dashed line is a Bonferroni correction, a p-value of 4.32×10^{-7}. The solid line is a suggestive line, which was calculated by removing SNPs with an r^2 of higher than 0.6, was 7.24×10^{-6}.

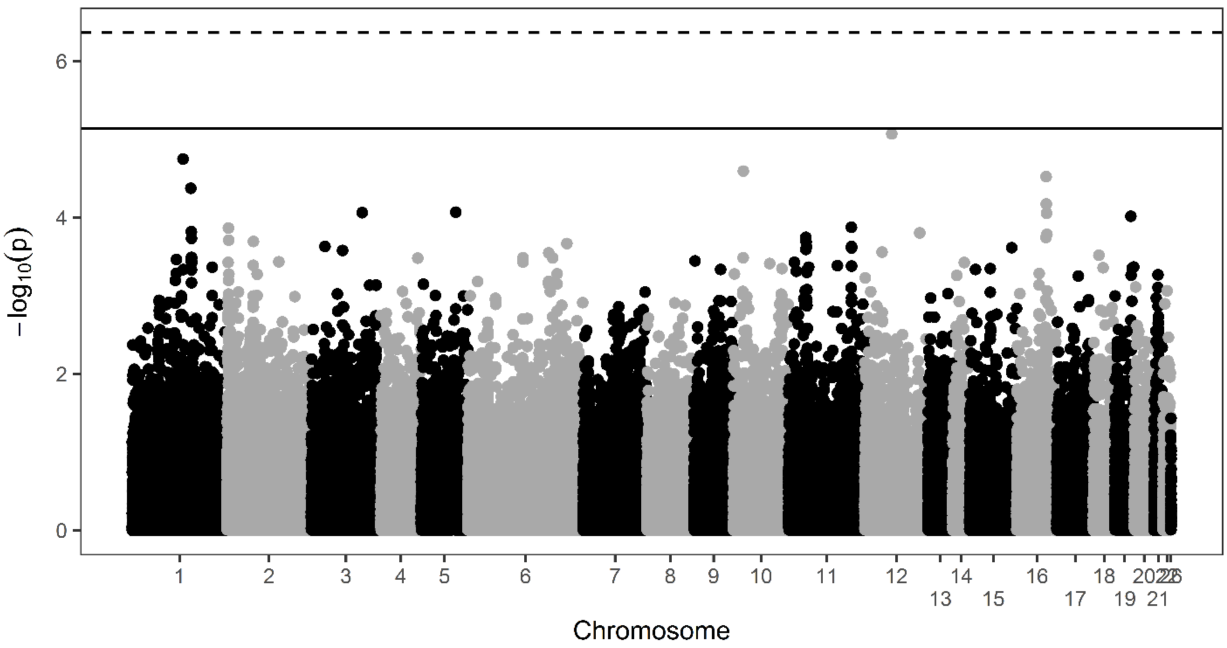

Figure S12. Manhattan plot of interaction term p-values for premedication diastolic blood pressure analysis.

Analysis of SNPs associated with median premedication diastolic blood pressure was conducted in an African American population from Vanderbilt's biobank, BioVU (n=2,577). Education and SNP main effects, as well as SNP x education interactions were included in this model. Education is coded as less than high school, high school and equivalent, and some college and above. Covariates included in the linear regression model of median premedication systolic blood pressure included age, age squared, sex, body mass index, smoking status, and African ancestry. The x-axis shows SNP position grouped by chromosome number. The y-axis shows the $-\log_{10}$ of the p-value for the SNP, which indicates that smaller p-values are higher on the axis. The dashed line is a Bonferroni correction, a p-value of 4.32×10^{-7}. The solid line is a suggestive line, which was calculated by removing SNPs with an r^2 of higher than 0.6, was 7.24×10^{-6}.

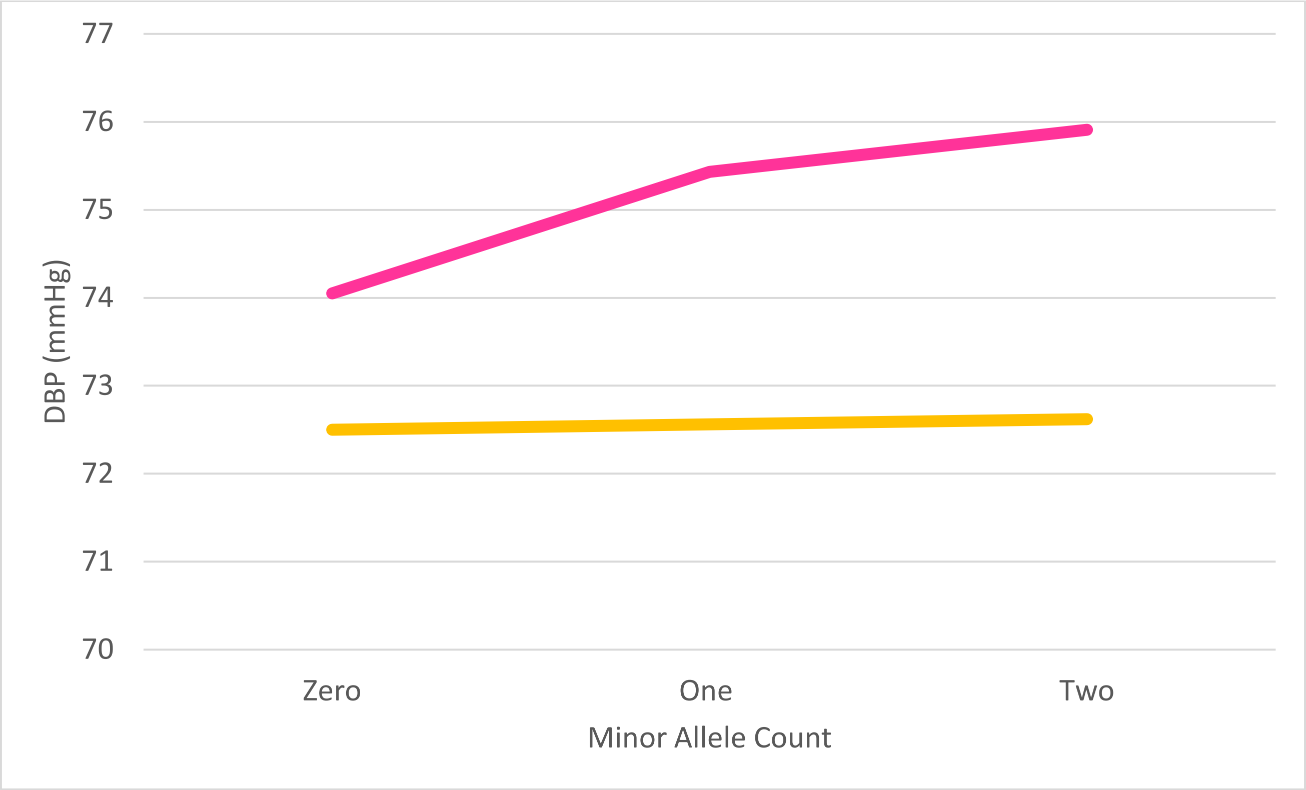

Figure S13. SNP x Education interaction affecting premedication diastolic blood pressure (DBP).

The pink line represents individuals with less than a high school degree. The orange line represents individuals with a high school degree and above. Individuals homozygous for the minor allele and having less than a high school education had higher diastolic blood pressure compared with 1) individuals homozygous for the minor allele and high school education or greater and 2) those not homozygous for the minor allele and less than a high school education.
